# Urotensin II Promotes Atherosclerosis in Cholesterol-Fed Rabbits

**DOI:** 10.1371/journal.pone.0095089

**Published:** 2014-04-18

**Authors:** Yafeng Li, Sihai Zhao, Yanli Wang, Yulong Chen, Yan Lin, Ninghong Zhu, Huadong Zheng, Min Wu, Daxing Cheng, Yandong Li, Liang Bai, Jianglin Fan, Enqi Liu

**Affiliations:** 1 Laboratory for Lipid Metabolism and Atherosclerosis, Xi'an Jiaotong University Cardiovascular Research Center, Xi'an, Shaanxi, China; 2 Laboratory Animal Center, Xi'an Jiaotong University School of Medicine, Xi'an, Shaanxi, China; 3 Department of Molecular Pathology, Interdisciplinary Graduate School of Medicine and Engineering, University of Yamanashi, Yamanashi, Japan; Brigham and Women's Hospital, Harvard Medical School, United States of America

## Abstract

Urotensin II (UII) is a vasoactive peptide composed of 11 amino acids that has been implicated to contribute to the development of cardiovascular disease. The purpose of this study was to investigate whether UII affects the development of atherosclerosis in cholesterol-fed rabbits. UII was infused for 16 weeks through an osmotic mini-pump into male Japanese White rabbits fed on a high-cholesterol diet. Plasma lipids and body weight were measured every 4 weeks. Aortic atherosclerotic lesions along with cellular components, collagen fibers, matrix metalloproteinase-1 and -9 were examined. Moreover, vulnerability index of atherosclerotic plaques was evaluated. UII infusion significantly increased atherosclerotic lesions within the entire aorta by 21% over the control (P = 0.013). Atherosclerotic lesions were increased by 24% in the aortic arch (P = 0.005), 11% in the thoracic aorta (P = 0.054) and 18% in the abdominal aorta (P = 0.035). These increases occurred without changes in plasma levels of total cholesterol, low-density lipoprotein cholesterol, high-density lipoprotein cholesterol, triglycerides or body weight. Immunohistochemical staining revealed that macrophages and matrix metalloproteinase-9 were significantly enhanced by 2.2-fold and 1.6-fold in UII group. In vitro studies demonstrated that UII up-regulated the expression of vascular cell adhesion protein-1 and intercellular adhesion molecule-1 in human umbilical vein endothelial cells, which was inhibited by the UII receptor antagonist urantide. In conclusion, our results showed that UII promotes the development of atherosclerotic lesions and destabilizes atherosclerotic plaques in cholesterol-fed rabbits.

## Introduction

Urotensin II (UII) is a vasoactive cyclic peptide composed of 11 amino acids that was originally identified in the spinal cord of teleost fish [1]. As a ligand, UII exerts its activity by binding to a G-protein coupled receptor named UT [2]. UII and the UII receptor UT are both highly expressed in the human cardiovascular system [3]. Plasma UII levels are elevated in various diseases, including essential hypertension, coronary heart disease, congestive heart failure, diabetes mellitus and renal failure [4]–[9]. Atherosclerosis is the primary cause of morbidity and mortality of cardiovascular diseases. Clinical studies have shown a positive correlation between increased plasma UII levels and the severity of atherosclerosis in coronary and carotid arteries [6], [10]. Studies using mouse models showed that UII may participate in the development of atherosclerosis [11], [12]. However, it is unclear whether UII affects atherosclerotic plaque stability. Rabbits are a suitable model for atherosclerosis research because of their rapid development of hyperlipidemia and atherosclerosis due to their sensitivity to dietary fat and cholesterol. In addition, rabbit atherosclerotic lesions resemble those observed in humans ranging from early stage lesions (fatty streaks) to complicated lesions (fibrous plaques). Finally, rabbits have lipoprotein profiles that are more similar to humans than those of mice [13]. In the current study, we were attempted to evaluate whether UII exerts any effects on the development of atherosclerosis in cholesterol-fed rabbits. Our results showed that chronic infusion of UII enhances aortic atherosclerotic lesions and affects the stability of the atherosclerotic plaques.

## Materials and Methods

### Animals

**Figure 1:**
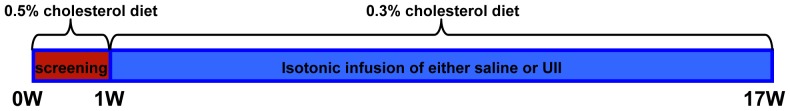
A schematic illustration of the experimental design.

Male Japanese White rabbits were provided by the Laboratory Animal Center of Xi'an Jiaotong University. All rabbits were initially screened for their response to cholesterol by feeding 0.5% cholesterol diet for one week (Figure 1). After that, rabbits whose plasma total cholesterol (TC) levels were between 200∼500 mg/dl, were selected for the current study. Rabbits were divided into two groups (vehicle and UII, n = 9 for each group) and were fed a cholesterol diet containing 0.3% cholesterol and 3% bean oil for 16 weeks. The rabbits were individually housed in metal cages in an air-conditioned rooms under a 12 h light/12 h dark cycle. Water was allowed *ad libitum*, and 100 g/day food was provided. The animal experiments were approved by the Laboratory Animal Administration Committee of Xi'an Jiaotong University and carried out according to the Guidelines for Animal Experimentation of Xi'an Jiaotong University and the Guide for the Care and Use of Laboratory Animals published by the US National Institutes of Health (NIH Publication NO. 85-23, revised 2011).

### UII infusion

Osmotic mini-pumps (Alzet Model 2006D, Durect Co., Cupertino, CA) were loaded with either UII (Shanghai Gl biochem Ltd, Shanghai, China) dissolved in isotonic saline or saline alone (vehicle). The doses of UII used in the current study were based on a previous report [14] and our own experiment. We used two doses of UII: 2.7 µg/kg/h (as low dose) and 5.4 µg/kg/h (as high dose). In the first experiment, we found that UII at low dose did not affect atherosclerosis (see Figure S1 and Figure S2), so we doubled the doses for the current experiment. Mini-pumps were implanted subcutaneously into the nape and replaced by new pumps every 6 weeks. Rabbits were anesthetized with a mixture of phenobarbital sodium (20–40 mg/kg body weight intravenously) and xylazine hydrochloride (0.2 ml/kg body weight intramuscularly).

### Biochemical analyses

Blood was collected via the auricular artery after fasting overnight every 4 weeks. The tubes of blood containing EDTA anticoagulant were stored on ice and centrifuged (2000 rpm for 15 min at 4°C) to obtain plasma. Plasma glucose, TC, triglycerides (TG), low-density lipoprotein cholesterol (LDL-C), and high-density lipoprotein cholesterol (HDL-C) were analyzed using commercial kits (Biosino Bio-technology & Science Inc., Beijing, China).

### Quantitative analysis of atherosclerotic lesions

Rabbits were sacrificed with an injection of phenobarbital sodium and xylazine hydrochloride. The aortic tree was isolated, opened longitudinally, fixed in 10% neutral buffered formalin, and stained with Sudan IV [15]. The area of the atherosclerotic lesion (Sudanophilic area) was measured using an image analysis software (Mitani Co., Tokyo, Japan) [15].

For the microscopic quantification of the lesion area, the aortic arch was cut into 8–10 sections. Sections were embedded in paraffin and cut into 5-µm thick serial sections, stained with hematoxylin and eosin (H&E), and elastica van Gieson (EVG). For microscopic evaluation of the cellular components, serial paraffin sections were immunohistochemically stained with antibodies (Abs) against macrophages (MΦ) (Dako Inc. Carpinteria, CA), smooth muscle α-actin (Thermo Fisher Scientific Inc. Rockford, IL), matrix metalloproteinase-1 (MMP-1) and MMP-9 as previously described [16]. In order to evaluate the collagen contents, the sections were stained with Masson's trichrome staining as previously described [17]. To evaluate the cellular proliferation and death in the lesions, we performed TUNEL staining using the *in situ* cell death detection kits (Promega, Madison, WI) and immunohistolchemical staining using the Ab against PCNA (Bioss, Beijing, China). TUNEL and PCNA positive cells were counted as previously reported [18].

To evaluate the plaque stability, we used frozen sections embedded in OCT compound and stained them for oil red O [11] and Masson's trichrome, MΦ and smooth muscle cells (SMCs). The plaque vulnerability was calculated by dividing the sum of area of MΦ and extracellular lipid deposits by sum of area for SMCs and collagen fibers as previously described by others [18].

### *In vitro* experiments

Human umbilical vein endothelial cells (HUVECs) were isolated from human umbilical cords using 0.1% (w/v) collagenase (Gibco/Invitrogen, Carlsbad, CA). HUVECs were cultured at 37°C in a humidified atmosphere of 5% CO_2_ and 95% air in M199 medium (Gibco/Invitrogen, Carlsbad, CA) supplemented with 20% fetal bovine serum (Gibco/Invitrogen, Carlsbad, CA), 100 U/ml penicillin (Life Technologies, Carlsbad, CA), 100 µg/ml streptomycin (Life Technologies, Carlsbad, CA), 100 µg/ml heparin sodium (Fluka/Sigma-Aldrich, St Louis, MO), and 1% (v/v) endothelial cell growth supplement (Sciencell Research Laboratories, Carlsbad, CA) [19]. The HUVECs were treated with UII (50 nM) alone or UII with UII receptor antagonist urantide (Shanghai Gl biochem Ltd., Shanghai, China) (30 nM) for 24 hours. Aliquots of 20 µg of cellular protein were separated by 10% SDS-PAGE and subjected to Western blotting with rabbit polyclonal Abs against human vascular cell adhesion molecule-1 (VCAM-1) (Epitomics, Burlingame, CA) and intercellular adhesion molecule-1 (ICAM-1) (Epitomics, Burlingame, CA). Umbilical cords were obtained from participants who provided informed consent. The protocol was approved by the Xi'an Jiaotong University Second Affiliated Hospital Ethics Committee. The investigation also adhered to the principles outlined in the Declaration of Helsinki for the use of human tissues or subjects.

### Statistical analysis

All data are expressed as the mean ±SEM. Statistical analyses were performed using either the Student's t-test with an equal F value or Welch's t test when the F value was not equal. P<0.05 was considered statistically significant.

## Results

### Plasma parameters and body weight

**Figure 2:**
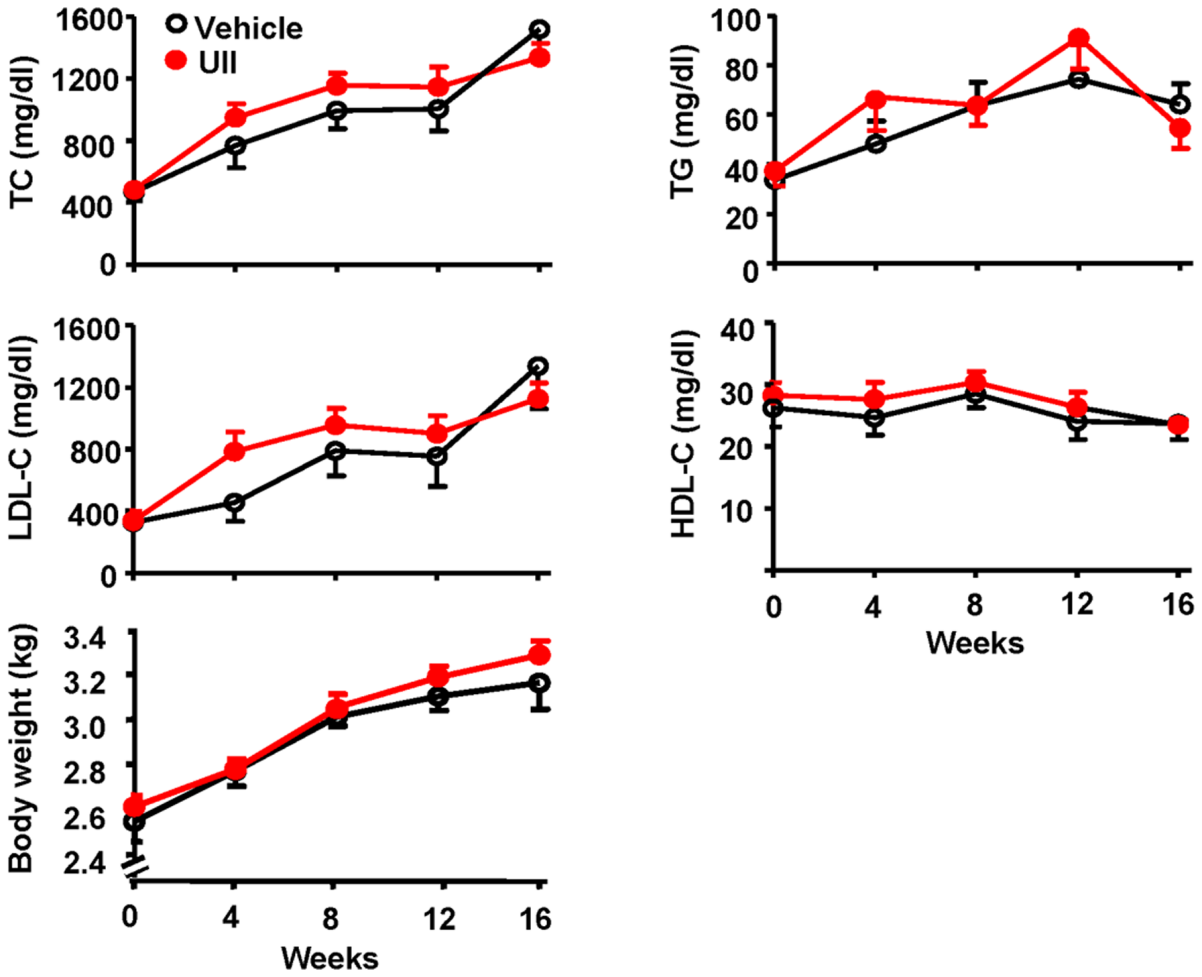
The plasma levels of total cholesterol (TC), triglycerides (TG), low density lipoprotein cholesterol (LDL-C), high density lipoprotein cholesterol (HDL-C) and body weigh. n = 9 for each group. Data are expressed as the mean ±SEM.

Rabbit plasma lipids were measured every four weeks. Chronic UII infusion did not alter plasma TC, LDL-C, HDL-C and TG levels (Figure 2).

Body weight, plasma glucose levels, systolic and diastolic blood pressure were also measured before and after UII infusion at 16 weeks. There is no significant difference between two groups (Figure 2 and Table.1).

**Table 1:** Main organs weight, blood pressure and plasma glucose at the end of experiments.

	Vehicle (n = 9)	UII (n = 9)
Heart weight (g)	6.9±0.2	8.0±1.0
Heart weight (g)/body weight (kg)	2.2±0.1	2.5±0.3
Liver weight (g)	142.9±13.9	160.6±7.8
Spleen weight (g)	2.7±0.6	2.0±0.1
Kidney weight (g)	17.4±1.0	18.6±0.4
Systolic blood pressure (mmHg)	144.7±4.1	150.1±2.7
Diastolic blood pressure (mmHg)	108.0±4.3	109.1±2.5
Plasma glucose (mg/dl)	233.8±21.2	242.1±15.5

Data are expressed as the mean ±SEM.

### Quantification of gross atherosclerotic lesions

**Figure 3:**
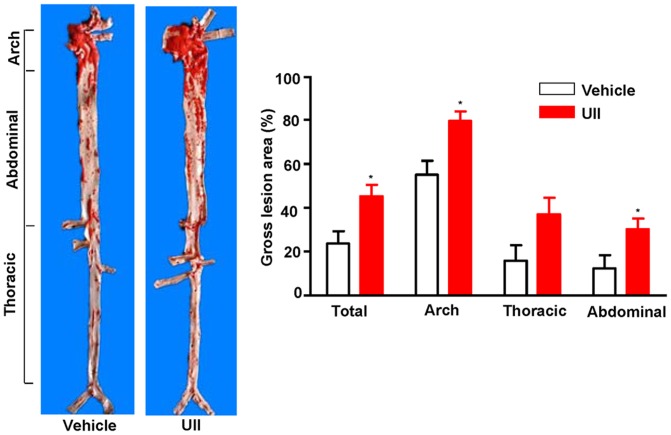
Representative picture of Sudan IV staining of aortas and quantitative analysis of lesion areas. n = 9 for each group. Data are expressed as the mean ±SEM. *P < 0.05 vs. vehicle.

The aortas were collected and stained with Sudan IV. The size of the atherosclerotic lesions were examined and quantified with an image analysis system. When compared to the vehicle group, the *en face* lesion area in the aortic trees of UII group was significantly increased by 21% (P = 0.013) in the total aorta, 24% (P = 0.005) in the aortic arch, 11% (P = 0.054) in the thoracic aorta and 18% (P = 0.035) in the abdominal aorta (Figure 3).

**Figure 4:**
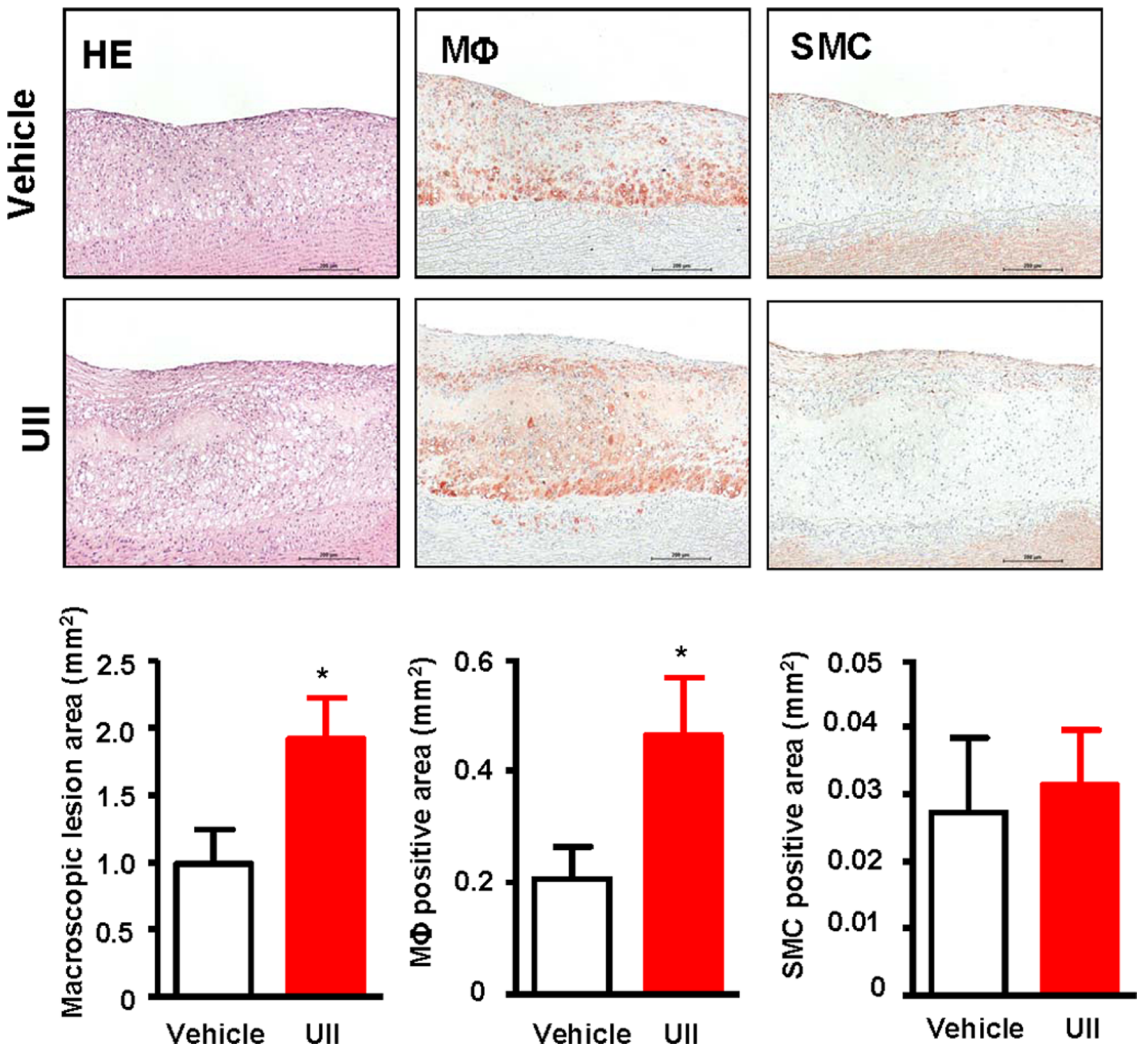
Representative micrographs of the intimal lesions and cellular components. Aortic sections were stained with H&E, or immunohistochemically stained with Abs against either MΦ and SMCs. Quantitative analysis of aortic arch lesion area and cellular composition of MΦ and SMCs is shown in the bottom. n = 9 for each group. Data are expressed as the mean ±SEM. *P <0.05 vs. vehicle.

Histological examination revealed that the aortic atherosclerotic lesions basically consisted of fatty streaks. Compared with the vehicle group, UII treatment significantly increased the microscopic intimal lesions in the aortic arch of by 1.9-fold (P = 0.033) (Figure 4). The MΦ positive area was increased by 2.2-fold (P = 0.045) but there was no difference in SMCs (p = 0.766) between the UII and vehicle groups (Figure 4).

**Figure 5:**
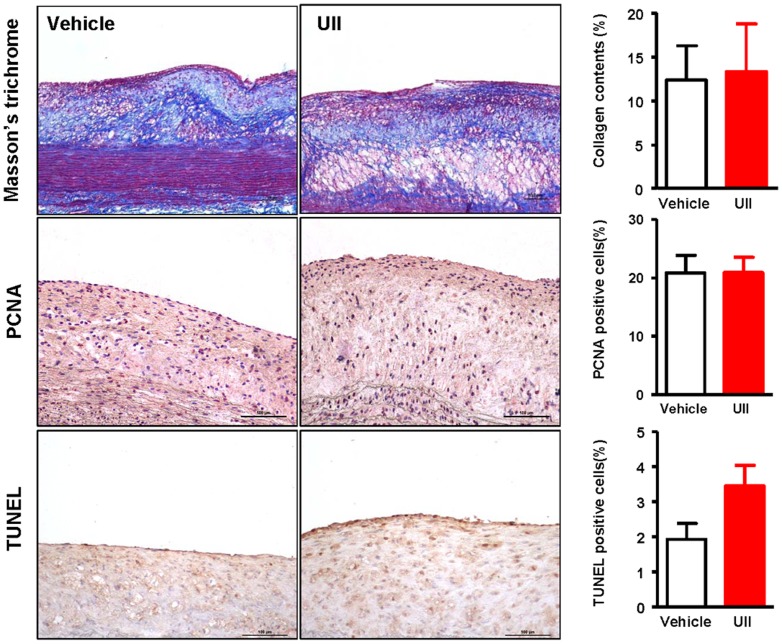
Representative micrographs of the intimal lesions. Aortic sections were stained with Masson's thichrome, PCNA and TUNEL and the ratio of each positive content to the lesion were calculated and showed in the right. n = 6 for each group. Data are expressed as the mean ±SEM.

We also evaluated the collagen contents and cellular proliferative state, and found that UII did not affect them but TUNEL-positive cells were increased compared to the vehicle group even though the difference did not reach statistical significance (Figure 5).

**Figure 6:**
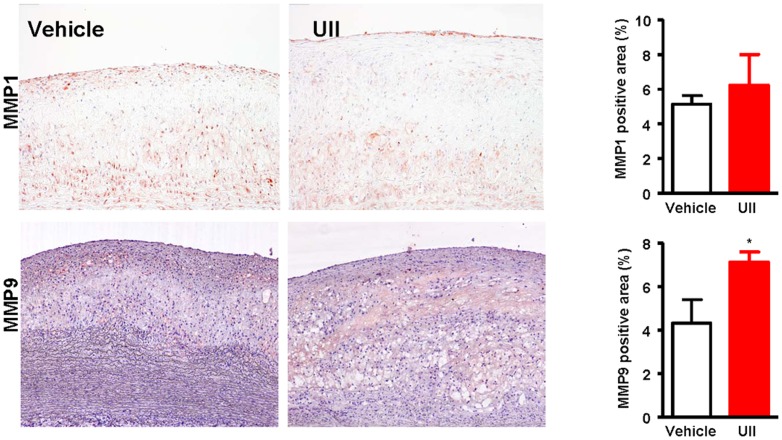
Representative micrographs of the intimal lesions. Aortic sections were immunohitochemically stained with MMP-1 and MMP-9. Quantitative analysis of MMP-1 and MMP-9 immunorecative proteins is shown in the right. n = 6 for each group. Data are expressed as the mean ±SEM. *P<0.05 vs. vehicle.

**Figure 7:**
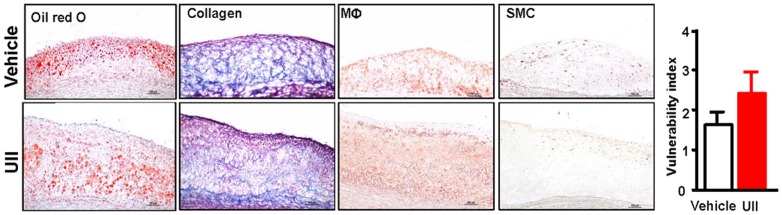
Representative micrographs of lesion components and vulnerability index. n = 3 for each group. Data are expressed as the mean ±SEM.

Finally, we examined the expression of MMP-1 and MMP-9 in the atherosclerotic lesions by immunohistochemical staining and found that MMP-9 but not MMP-1 immunorecative proteins were increased by 1.6-fold (P = 0.038) compared with vehicle group (Figure 6). To investigate whether increased macrophage number and MMP-9 expression had any relationship with the plaque stability, we further evaluated the plaque vulnerability index and found that UII group had higher vulnerability index than the vehicle group but the difference did not reach statistical significance (P = 0.353) (Figure 7).

### Effects of UII on adhesion molecules

**Figure 8:**
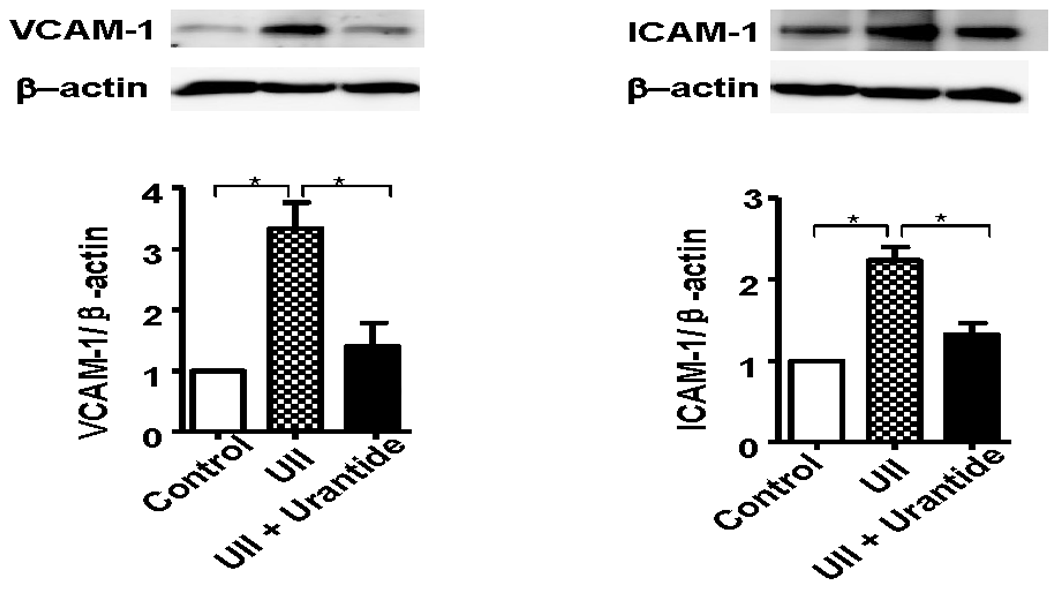
Effects of UII on VCAM-1 and ICAM-1 protein expression in HUVECs by Western blotting. n = 3 for each group. Data are expressed as the mean ±SEM. *P<0.05.

To determine the effects of UII on the expression of vascular adhesion molecules of HUVECs, we performed the in vitro experiments. As shown in Figure 8, UII treatment significantly increased the expression of VCAM-1 (3.3-fold) and ICAM-1 (2.2-fold) compared to the vehicle. Furthermore, the increased expression of VCAM-1 and ICAM-1 was inhibited by the UII receptor antagonist urantide (P = 0.005).

## Discussion

The present study demonstrated that UII enhances aortic atherosclerotic lesions by increasing macrophage infiltration and the expression of MMP9 in atherosclerotic lesion in cholesterol-diet-fed rabbits. Our data also showed that UII may increase the susceptibility to the plaque rupture because the lesions of UII group contained more macrophages and MMP-9 than the control group. This pro-atherogenic effect of UII was independent of any change in plasma lipids because the TC, LDL-C and TG levels in UII group did not differ from the vehicle group. Our findings are consistent with a previous study in which chronic UII infusion leads to increased aortic atherosclerotic lesions in apoE knockout mice [11]. UII was reported to play an important role in the regulation of macrophage-derived foam cell formation. Watanabe et al. reported that UII accelerates the formation of macrophage-derived foam cells by upregulating Acyl-coenzyme A:cholesterol acyltransferase-1 expression via the pathway of UT receptor/G-protein/c-Src/PKC/MEK and ROCK [20]. Shiraishi and coworkers also demonstrated that UII infusion results in a significant increase in ox-LDL-induced cholesteryl ester accumulation in macrophages [11]. Moreover, our study showed for the first time that UII increases macrophage-derived foam cells in the lesions of cholesterol-fed rabbits. The possible mechanism for this phenomenon may be related to the fact that UII increases the expression of adhesion molecules (VCAM-1 and ICAM-1) on the endothelial cells by which more monocytes were recruited into the intima. Enhancement of intimal MΦ accumulation may increase the vulnerability of the plaques or make the plaques prone to rupture, leading to thrombosis [21]. It should be pointed out that MMP-9, an important regulator of the plaque stability was also increased in the UII group. It is currently unknown whether increased MMP-9 was caused by the increased number of MΦ or whether UII has any direct effect on MMP-9 expression by MΦ or both. It will be also important to investigate in future whether UII has any effects on other enzymes in the lesions such as cathepsins that are involved in the plaque stability [22]–[23]. Nevertheless, all these features suggest that increased plasma levels of UII can constitute another risk factor for the plaque rupture.

In the current study, we found that the doses of UII are important for the pro-atherogenic effects because the low dose of UII did not have any effects on the development of atherosclerosis in cholesterol-fed rabbits, suggesting that there is a threshold in the plasma UII determining whether UII is pro-atherogenic. Vascular SMC proliferation also plays a crucial role in the progression of atherosclerosis. A line of evidence suggests that UII induces SMC proliferation [24]–[26]. However, we did not find a significant difference in the SMCs of the atherosclerotic lesions between UII and vehicle groups. It is not known whether UII has any effects on the advanced lesions [27] since the cholesterol-fed rabbits may have more early stage atherosclerotic lesions. Cheung et al. reported that the plasma UII level is positively correlated with body weight in the Hongkong Chinese population [4]. Although UII seems to play an important role in the control of body weight [12], in our study, we did not find significant body weight gain in the UII compared to the vehicle group, suggesting that UII may not directly cause body weight increase.

In summary, the results of the present study support a role for UII in promoting the progression of atherosclerosis by increasing the accumulation of MΦ in the atherosclerotic lesions. UII increases the expression of VCAM-1 and ICAM-1 in vascular endothelial cells. Moreover, UII may destablize the atherosclerotic plaques.

## Supporting Information

Figure S1 **Low dose effects of UII on the development of atherosclerosis in cholesterol-fed rabbits as described in the Materials.** Sudan IV staining of aortas and quantitative analysis of lesion areas. n = 8 for each group. Data are expressed as the mean ±SEM.
(TIF)

Figure S2 **Representative micrographs of the intimal lesions and cellular components.** Aortic sections were stained with H&E, or immunohistochemically stained with Abs against either MΦ or SMCs. Quantitative analysis of aortic arch lesion area and cellular contents of MΦ and SMCs is shown in the bottom. n = 8 for each group. Data are expressed as the mean ±SEM.
(TIF)
