# Supplementary figures and images for: Urotensin II Promotes Atherosclerosis in Cholesterol-Fed Rabbits
Source: PLoS One. 2014 Apr 18;9(4):e95089. doi: 10.1371/journal.pone.0095089 (PMC3991611; doi:10.1371/journal.pone.0095089)

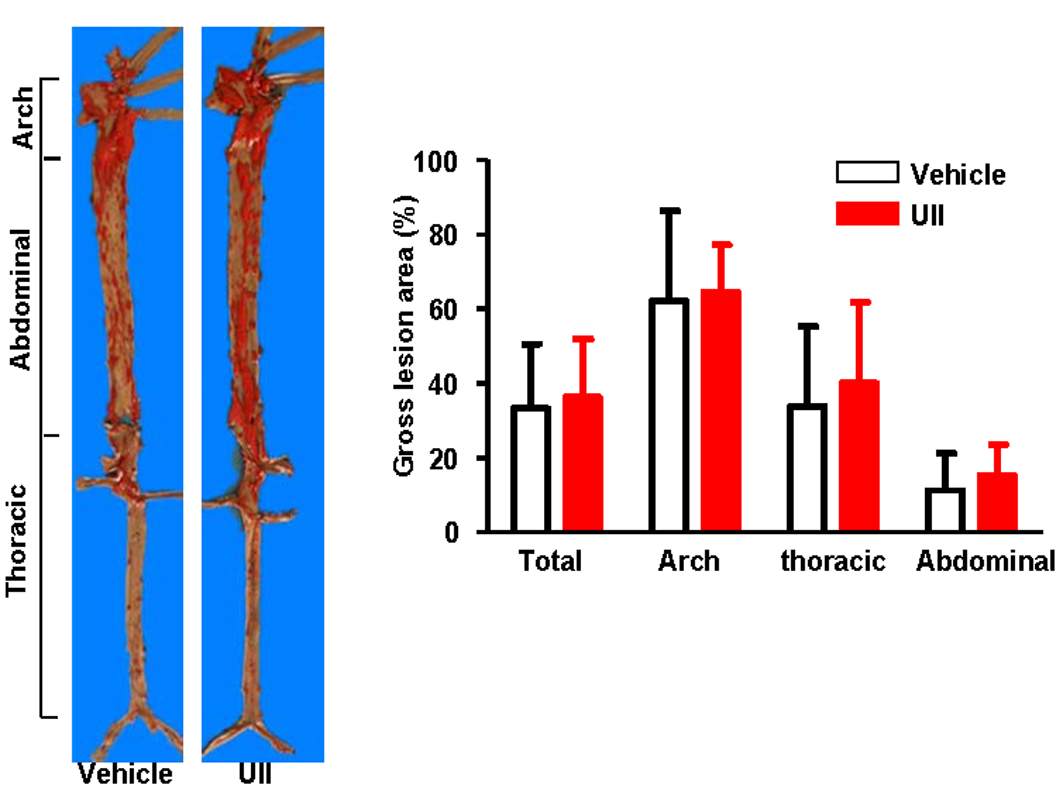

Supplement: Figure S1 — Low dose effects of UII on the development of atherosclerosis in cholesterol-fed rabbits as described in the Materials. Sudan IV staining of aortas and quantitative analysis of lesion areas. n = 8 for each group. Data are expressed as the mean ±SEM. (TIF) [file pone.0095089.s001.tif]

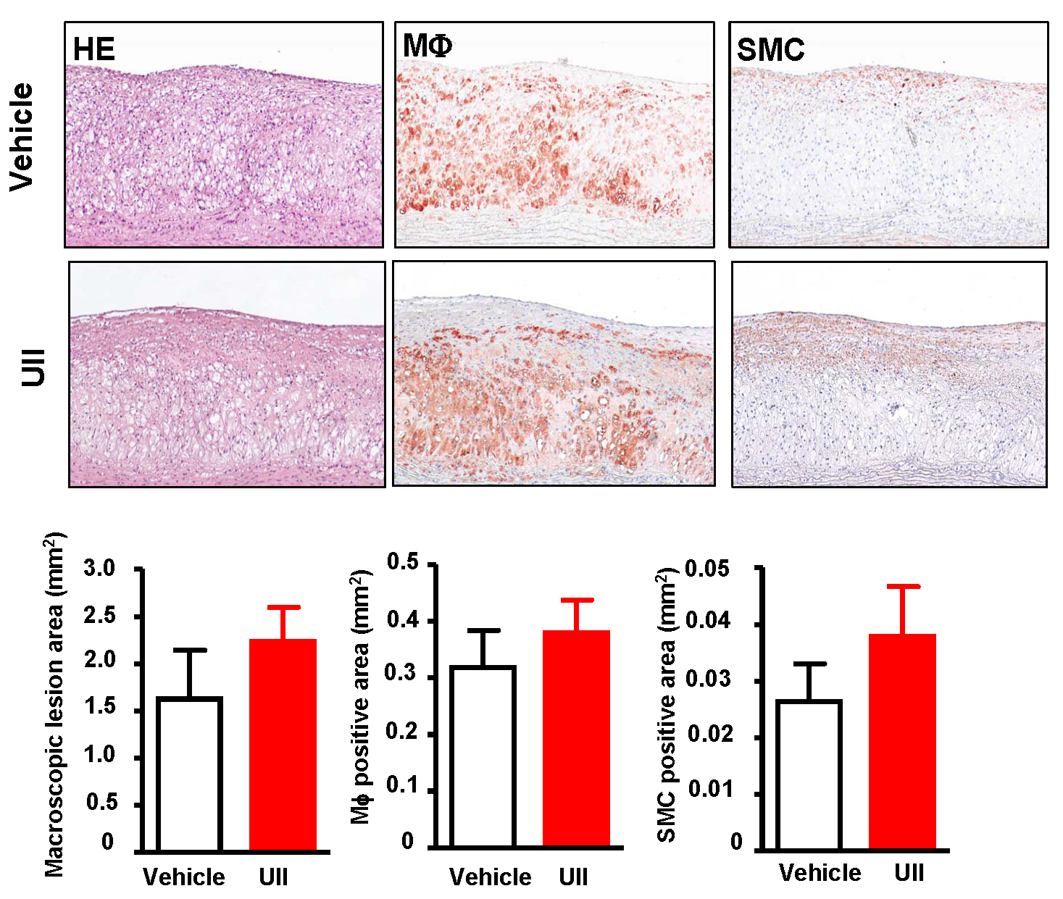

Supplement: Figure S2 — Representative micrographs of the intimal lesions and cellular components. Aortic sections were stained with H&E, or immunohistochemically stained with Abs against either MΦ or SMCs. Quantitative analysis of aortic arch lesion area and cellular contents of MΦ and SMCs is shown in the bottom. n = 8 for each group. Data are expressed as the mean ±SEM. (TIF) [file pone.0095089.s002.tif]
